# Long-Term Burden of Increased Body Mass Index from Childhood on Adult Dyslipidemia: The i3C Consortium Study

**DOI:** 10.3390/jcm8101725

**Published:** 2019-10-18

**Authors:** Yinkun Yan, Lydia A. Bazzano, Markus Juonala, Olli T. Raitakari, Jorma S. A. Viikari, Ronald Prineas, Terence Dwyer, Alan Sinaiko, Trudy L. Burns, Stephen R. Daniels, Jessica G. Woo, Philip R. Khoury, Elaine M. Urbina, David R. Jacobs, Tian Hu, Julia Steinberger, Alison Venn, Wei Chen

**Affiliations:** 1 Department of Epidemiology, School of Public Health & Tropical Medicine, Tulane University, New Orleans, LA 70112, USA; ykyan2011@163.com (Y.Y.); lbazzano@tulane.edu (L.A.B.); 2 Department of Medicine, University of Turku, Turku 20500, Finland; mataju@utu.fi (M.J.); jorvii@utu.fi (J.S.A.V.); 3 Division of Medicine, Turku University Hospital, Turku 20521, Finland; 4 Murdoch Children’s Research Institute, Parkville, Victoria 3052, Australia; 5 Research Centre of Applied and Preventive Cardiovascular Medicine, University of Turku, Turku FI-20520, Finland; olli.raitakari@utu.fi; 6 Department of Clinical Physiology and Nuclear Medicine, Turku University Hospital, Turku 20521, Finland; 7 Division of Public Health Sciences, Wake Forest University School of Medicine, Winston-Salem, NC 27101, USA; rprineas@wakehealth.edu; 8 George Institute, University of Oxford, Oxford OX1 2BQ, UK; terence.dwyer@georgeinstitute.ox.ac.uk; 9 Department of Pediatrics, University of Minnesota, Minneapolis, MN 55455, USA; sinai001@umn.edu; 10 Department of Epidemiology, College of Public Health, University of Iowa, Iowa City, IA 52246, USA; trudy-burns@uiowa.edu; 11 Department of Pediatrics, University of Colorado School of Medicine, Aurora, CO 80045, USA; Stephen.Daniels@childrenscolorado.org; 12 Division of Biostatistics and Epidemiology, Cincinnati Children’s Hospital Medical Center, Cincinnati, OH 45229, USA; Jessica.Woo@cchmc.org; 13 Department of Pediatrics, University of Cincinnati College of Medicine, Cincinnati, OH 45229, USA; Phil.khoury@cchmc.org (P.R.K.); Elaine.Urbina@cchmc.org (E.M.U.); 14 The Heart Institute, Cincinnati Children’s Hospital Medical Center, Cincinnati, OH 45229, USA; 15 Division of Epidemiology and Community Health, School of Public Health, University of Minnesota, Minneapolis, MN 55455, USAhuxxx405@umn.edu (T.H.); 16 Division of Pediatric Cardiology, Department of Pediatrics, Medical School, University of Minnesota, Minneapolis, MN 55455, USA; Stein055@umn.edu; 17 Menzies Institute for Medical Research, University of Tasmania, Hobart TAS 7000, Australia; Alison.Venn@utas.edu.au

**Keywords:** body mass index, dyslipidemia, longitudinal study, childhood

## Abstract

**Background:** Data are limited regarding the association of cumulative burden and trajectory of body mass index (BMI) from early life with adult lipid disorders. **Methods:** The study cohort consisted of 5195 adults who had BMI repeatedly measured 4 to 21 times from childhood and had blood lipid measurements of low-density lipoprotein cholesterol (LDL-C), high-density lipoprotein cholesterol (HDL-C), and triglycerides (TG) and information on lipid-lowering medications in the last adult survey. The area under the curve (AUC) was calculated as a measure of long-term burden (total AUC) and trends (incremental AUC) of BMI. **Results:** Participants with dyslipidemia, high LDL-C, low HDL-C and high TG had consistently and significantly higher BMI levels from childhood to adulthood compared to those with normal lipid levels. After adjusting for age, race, sex, and cohort, increased risk of adult dyslipidemia was significantly associated with higher values of childhood BMI, adulthood BMI, total AUC and incremental AUC, with odds ratio (95% confidence interval) = 1.22 (1.15–1.29), 1.85 (1.74–1.97), 1.61 (1.52–1.71), and 1.59 (1.50–1.69), respectively, and *p* < 0.001 for all. The association patterns were similar in most race–sex subgroups. **Conclusions:** Adults with dyslipidemia versus normal lipid levels have consistently higher levels and distinct life-course trajectories of BMI, suggesting that the impact of excessive body weight on dyslipidemia originates in early life.

## 1. Introduction

Dyslipidemia has been a major public health problem [1,2]. Clinical and epidemiologic studies have demonstrated that lipid disorders are associated with insulin resistance and predict increased risk of cardiovascular mortality and morbidity [3]. Obesity is a pandemic of the modern world [4], and is intimately associated with dyslipidemia for both youth and adults [5,6,7]. Many longitudinal cohorts followed since childhood have shown that early-life body mass index (BMI) is associated with an adult adverse lipid profile [8,9,10,11,12]. Data, however, are limited regarding the influence of long-term cumulative burden of excessive body weight from childhood on adult dyslipidemia.

BMI changes dramatically with age but tends to track in the same ranking over time. Higher BMI in childhood strongly predicts increased risk of adult obesity, suggesting that adult obesity originates in early life [13,14]. Nonlinear growth trajectories of BMI from childhood to midlife have been characterized in the Bogalusa Heart Study cohort [15]. During the past couple of decades, a statistical methodology, group-based trajectory modeling, has been increasingly applied to map the developmental course of changes with age in cardiovascular risk factors. Life-long follow-up cohort studies have demonstrated that trajectory group membership of longitudinal BMI growth during different age periods is associated with cardiovascular disease and cardiometabolic risk, including lipid disorders [16,17,18,19].

In previous studies using the group-based trajectory model [16,17,18,19], the membership of distinct groups was used as a risk factor variable to assess its association with outcomes. Individual-level trajectory parameters of longitudinal changes in BMI would provide detailed information on the patterns of change in body weight in a particular age period from early life in relation to subsequent health outcomes. However, no studies have examined whether adults with and without dyslipidemia have distinct life-course BMI trajectories from childhood. Utilizing pooled data from the International Childhood Cardiovascular Cohort (i3C) Consortium, we aimed to characterize the individual-specific life-course growth trajectories and cumulative burden of BMI from childhood to adulthood and examine their associations with adult dyslipidemia.

## 2. Materials and Methods

### 2.1. Study Cohort

Detailed descriptions for the i3C Consortium have been published elsewhere [20,21]. In brief, the i3C Consortium included seven longitudinal cohort studies initiated in childhood from the US, Finland and Australia. In this study, we included participants from the i3C Consortium who were examined for weight and height at least 2 times in childhood (3–19 years) and at least 2 times in adulthood (20–52 years) and had blood lipid measurements of low-density lipoprotein cholesterol (LDL-C), high-density lipoprotein cholesterol (HDL-C) and triglycerides (TG), and information on lipid-lowering medications in the last adult survey. According to these inclusion criteria, the final study cohort consisted of 5195 participants from five of the seven cohorts, i.e., the Bogalusa Heart Study (BHS), the Muscatine Study (MUSC), the National Heart, Lung and Blood Institute Growth and Health Study (NGHS) and the Prevention of High Blood Pressure in Children Study (PHBPC) in the US, and the Cardiovascular Risk in Young Finns Study (YFS) in Finland. Detailed descriptions of these cohorts are provided in the Supplementary Materials. The average number of repeated measurements for weight and height from childhood to adulthood was 8.8 (range = 4–21).

All participants or their legal guardians at each examination provided written informed consent. The study protocols were approved by the respective Institutional Review Boards.

### 2.2. Examinations

Detailed study characteristics and examination methods for the five cohorts have been previously described [20,21,22,23,24,25]. According to the Third Report of the National Cholesterol Education Program Expert Panel on Detection, Evaluation and Treatment of High Blood Cholesterol in Adults [1], high LDL-C, low HDL-C and high TG were defined as ≥160 mg/dL, ≤40 mg/dL, ≥200 mg/dL, respectively, or taking lipid-lowering medications. Dyslipidemia was defined as the presence of any of high LDL-C, low HDL-C and high TG. There were 235 adult participants (4.5%) who took lipid-lowering medications.

### 2.3. Statistical Methods

Parameters of nonlinear growth curves of BMI from childhood to adulthood were estimated using a random-effects mixed model by SAS proc MIXED, as previously reported [26,27]. The mixed model incorporates fixed and random effects and allows the intercept, linear and nonlinear parameters to vary from individual to individual. The random effect coefficients represent deviations of BMI for individuals from the fixed-effect parameters. In addition, the mixed model allows for repeated measurements and different numbers of unequally spaced observations across individuals. The model computes maximum-likelihood estimates of growth curve parameters, including fixed-effect parameters (for a group) and random-effect parameters (individual specific). By combining these two types of parameters, 5195 sets of growth curve parameters were generated for each of 5195 participants of the study cohort. The model selection was based on the Akaike’s information criterion (AIC) and *p*-values of the independent variable (age) at the significance level of 0.05. Age and its higher-order terms were included one by one for model building. The higher-order terms of age were not included in the model if they were not significant, or made lower-order terms not significant, or did not improve the goodness-of-fit of the model based on AIC values. Cubic curves were fitted for BMI in race–sex groups:

$${\mathrm{BMI}}_{i}\;=\;(\beta_{0}\;+\;b_{0i})\;+\;(\beta_{1}\;+\;b_{1i})\;\mathrm{age}\;+\;(\beta_{2}\;+\;b_{2i})\;{\mathrm{age}}^{2}\;+\;(\beta_{3}\;+\;b_{3i})\;{\mathrm{age}}^{3}\;+\;ɛ$$

where β = (β_0_, β_1_, β_2_, β_3_)’ is a vector of fixed-effect parameters, b = (b_0_, b_1_, b_2_, b_3_)’ is a vector of random-effect parameters, and ɛ is an unknown error term. Age was centered to the mean age (22.1 years) to reduce the collinearity of age with its higher-order terms. The term age^2^ was divided by 10 and age^3^ by 20 to improve model fitting.

Long-term burden and trends of BMI were measured as the area under the curve (AUC). As shown in Supplemental Figure S1, the AUC was calculated as the integral of the curve parameters during the follow-up period for each participant. Because participants had different follow-up periods, the AUC values were divided by the number of follow-up years and used for the subsequent association analyses. Total AUC can be considered a measure of a long-term cumulative burden; incremental AUC, determined by within-subject variations, represents a combination of linear and nonlinear longitudinal trends.

Multivariable logistic regression analyses were performed to examine the association of dyslipidemia with childhood values (the first survey), adulthood values (the last survey), total and incremental AUC values of BMI, adjusted for adult age, race, sex and cohort. Four dichotomous dummy variables were created for the five cohorts. Prior to the regression analysis, childhood and AUC values of BMI were adjusted for childhood age and average age, respectively, using regression residual analyses in race–sex groups by cohort and then standardized by Z-transformation (mean = 0, SD = 1). In addition, for regression residual analyses of incremental AUC, baseline values of childhood BMI were included in the models for adjustment to control the regression-to-the-mean bias.

To examine whether there is an independent effect of childhood BMI on adult lipid disorders, the risk of dyslipidemia was examined in logistic regression models in four groups divided by the combination of low and high BMI in childhood/adulthood (low/low, high/low, low/high and high/high) using the low/low group as reference. Low and high BMI was defined by its race-, sex- and age-specific medians in childhood and 25.0 kg/m^2^ in adulthood as cut-offs. In addition, we also constructed BMI burden using all BMI values before the last adult BMI and linked the BMI burden to adult dyslipidemia by including the last adult BMI as a fixed effect in the association analysis models.

## 3. Results

The characteristics of study participants in total and five cohorts are shown in Table 1. The current study included 5195 participants, including 4212 (80.1%) whites and 2069 (39.8%) males, with mean (±SD) ages of 10.1 ± 3.2 years in the first childhood survey and 37.7 ± 7.2 years in the last adult survey. The mean length of follow-up was 27.3 ± 7.0 years. The prevalence of dyslipidemia in the total cohort was 36.9%, with BHS having the highest prevalence (45.7%) and NGHS having the lowest prevalence (22.8%). The prevalence of high LDL-C, low HDL-C and high TG in the total cohort was 14.9%, 26.1% and 13.8%, respectively. The overall prevalence of combined overweight and obesity was 31.5% and 30.8%, respectively. The comparison of study variables between the included and excluded participants and the characteristics of the study cohorts by race and sex are shown in Supplemental Tables S1 and S2, respectively.

**Figure 1:**
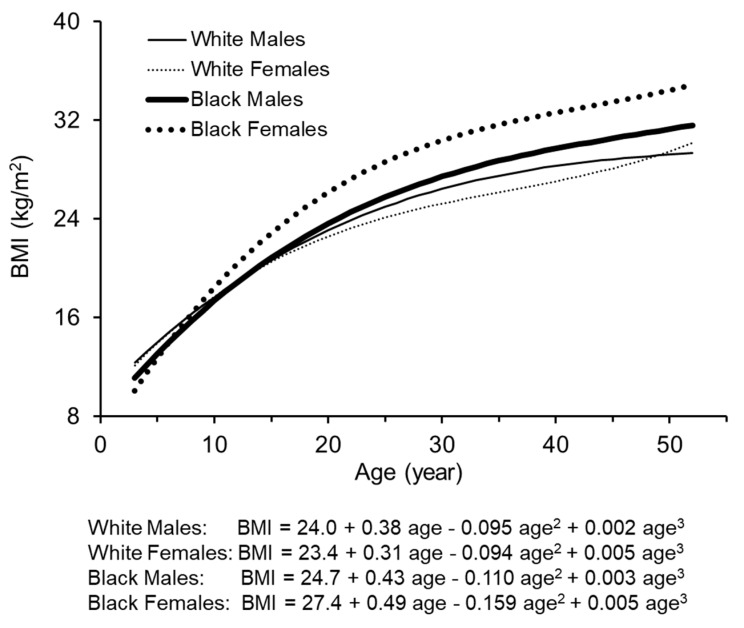
Growth curves of body mass index (BMI) by race and sex.

Figure 1 presents longitudinal trajectories of BMI from childhood to adulthood by race and sex. Black females had the fastest increase and highest levels from childhood to adulthood followed by black males, white males and white females. The growth curve of BMI in black females was separated from other groups around 10 years and beyond. The other three groups had similar trajectories until around 20 years of age; thereafter, white females tended to have a slower rate of increase and lower levels. Curve parameters (β_0_ + b_0_, β_1_ + b_1,_ β_2_ + b_2_ and β_3_ + b_3_) for BMI were all significantly different from 0.

**Figure 2:**
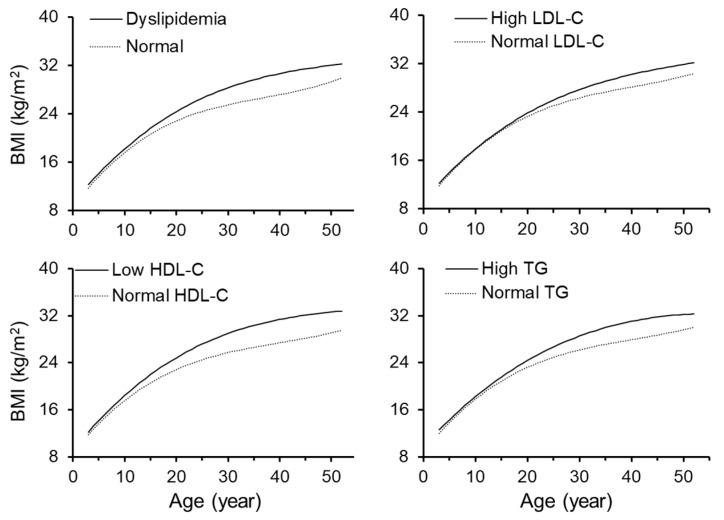
Growth curves of BMI in groups classified by dyslipidemia groups. BMI = body mass index; LDL-C = low-density lipoprotein cholesterol; HDL-C = high-density lipoprotein cholesterol; TG = triglycerides.

Figure 2 presents growth curves of BMI from childhood to adulthood in participants with abnormal versus normal lipid levels. Participants with dyslipidemia, high LDL-C, low HDL-C and high TG had consistently higher BMI levels from childhood to adulthood compared to those with normal lipid levels. The differences in BMI levels between the two comparison groups became greater with increasing age. Growth curves of BMI in adult dyslipidemia groups by race and sex were presented in Supplemental Figure S2.

Table 2 presents detailed information on curve parameters in participants with abnormal versus normal lipid levels. Adults with dyslipidemia compared to those with normal lipid levels had significantly greater values of β_0_ + b_0_, β_1_ + b_1_ and β_2_ + b_2_, but lower β_3_+b_3_. Trends in the differences were similar between abnormal and normal groups of LDL-C, HDL-C and TG, except for the difference in β_2_ + b_2_ between low and normal HDL-C groups.

Table 3 presents the odds ratios (ORs) for adult dyslipidemia in association with BMI in terms of childhood and adulthood, as well as total and incremental AUC values, adjusted for adult age, race, sex and cohort in separate regression models. All BMI values were associated with increased risk of adult dyslipidemia, high LDL-C, low HDL-C and high TG (*p* < 0.01 for all). Adult BMI had the highest OR (95% confidence interval) of 1.85 (1.74–1.97) for adult dyslipidemia, which was significantly greater than 1.22 (1.15–1.29), 1.61 (1.52–1.71) and 1.59 (1.50–1.69) for childhood BMI, total AUC and incremental AUC, respectively. Most of the associations remained significant in stratified analyses by race and sex (Supplemental Table S3). For white participants, females versus males had stronger associations of childhood BMI with dyslipidemia, high LDL-C and low HDL-C, and total AUC with low HDL-C, but a weaker association of adulthood BMI with high TG; no sex difference was found for black participants. Race differences in ORs for dyslipidemia (whites > blacks) were significant in females, with the exception of childhood BMI. Whites versus blacks had stronger associations of adult BMI with high TG. The total AUC-low HDL-C association was stronger in whites than in blacks in females.

Supplemental Table S4 shows the associations of BMI measures with dyslipidemia by adult age groups. All the ORs were significant except for childhood BMI in the age groups of 36–40, 41–45 and ≥46 years. There were no significant differences in strengths of the associations measured as OR values between adult age subgroups.

Supplemental Figure S3 shows ORs for lipid disorders according to childhood and adulthood BMI status after adjusting for race, sex, age and cohort. Compared with individuals with normal BMI consistently from childhood to adulthood (the low/low group), those in the high/high and low/high groups had significantly higher risks of dyslipidemia, high LDL-C, low HDL-C and high TG in adulthood. In addition, individuals with high childhood BMI but normal adult BMI had similar ORs for dyslipidemia, high LDL-C and low HDL-C and a lower OR for high TG.

Supplemental Table S5 shows ORs of BMI burden for adult dyslipidemia with and without including the last adult BMI in the model. After adjusting for race, sex, age and cohort, BMI burden from childhood was positively associated with increased risk of adult dyslipidemia in model 1s (*p* < 0.05 for all). When BMI burden and the last adult BMI were both included in the model (model 3s), the associations of BMI burden with dyslipidemia, high LDL-C, low HDL-C and high TG became negative (ORs < 1, *p* < 0.05 for all), and the ORs of the last adult BMI were greater than 1 (*p* < 0.05 for all).

## 4. Discussion

Using pooled data on repeated BMI measurements from childhood to midlife age from five longitudinal cohorts in the i3C Consortium, we characterized nonlinear BMI growth trajectories from childhood by race and sex. Adults with dyslipidemia had higher long-term BMI levels and greater linear slopes and change in slopes compared to those with normal lipid levels. Childhood BMI, adulthood BMI, long-term burden and increasing trends (AUCs) were positively and significantly associated with increased risk of adult dyslipidemia. These findings suggest that the influence of high BMI on the development of dyslipidemia begins at early life stages.

The strong relationship between obesity and adverse lipid profiles, including high LDL-C, low HDL-C and high TG, in both youth and adults has been documented in numerous cross-sectional studies [5,7]. Intervention studies have shown that weight loss through lifestyle changes has a significant desirable effect on lipid levels [28,29]. Longitudinal studies have reported that increased childhood BMI at a single time point is positively associated with cardiometabolic risk including lipid disorders in later life [8,9,10,11,12]. A previous i3C Consortium analysis has demonstrated that increased childhood BMI affects adult lipid profiles through adult obesity [30]. The influence of life-long cumulative burden of excessive adiposity on the development of dyslipidemia has not been adequately described. By taking advantage of multiple repeated measurements of BMI in longitudinal cohorts of the i3C Consortium, we found that high values of childhood BMI and its long-term cumulative burden measured as total AUC predicted increased risk of dyslipidemia in middle-aged adults. By comparing the growth curves of BMI, the difference in BMI levels between the normal and abnormal lipids groups became greater with increasing age, indicating that the effect of excessive body weight on dyslipidemia is accumulative and exacerbated during a person’s lifetime. In addition, we found that the associations of BMI with low HDL-C and high TG were stronger than the association for high HDL-C, which was consistent with previous studies [31,32]. Studies have shown that obesity leads to lipid metabolism abnormality, mainly due to free fatty acid release in excess of metabolic demand [31].

During the last two decades, group-based trajectory modeling has been widely used to characterize trajectory patterns of repeated measures of BMI over time. Several longitudinal studies that began in early life have reported that BMI trajectories from childhood are associated with increased risk of cardiovascular disease, type 2 diabetes, insulin resistance and cardiometabolic risk in adulthood [16,17,18,19]. In the Young Finns Study, six discrete groups of long-term BMI trajectories from childhood were identified, and individuals who had a BMI at high levels, over time, had increased cardiometabolic risk (hypertension, high LDL-C, low HDL-C and high TG) in midlife [16]. Group-based trajectory modeling was developed to generate the group average of curve parameters and use group membership as a predictor variable. In the current study, incremental AUC was calculated using the combination of linear and nonlinear BMI growth curve parameters to measure the long-term trends for each individual. We found a significant association of increasing trends in BMI measured as incremental AUC with adult dyslipidemia.

The growth curve parameters estimated in the random-effects model in this study provided detailed information on individual-specific parameters in specific age periods, as shown in Figure 1. β_0_ + b_0_ is the intercept (the level of BMI at age of 22.1) because age was centered at its sample mean value; β_1_ + b_1_ describes linear slopes (the tangent lines) at the age point of 22.1; the negative β_2_ + b_2_ from 3 to ~40 years shows that linear slopes at age points become smaller and smaller in this age period; and β_3_ + b_3_ reflects that β_2_ + b_2_ changes from negative to positive at inflection points which vary between 37 and 52 years by race–sex groups. In this study, participants with dyslipidemia had higher long-term levels and steeper rates of increase in BMI than those with normal lipids. The smaller absolute values of the negative β_2_ + b_2_ in dyslipidemic adults indicated that the linear slopes of BMI at age points from childhood in this group were greater than the normal lipid group. The lower values of β_3_ + b_3_ among dyslipidemic adults, as shown in Table 2, might be affected by the regression-to-the-mean phenomenon. BMI changes dramatically with age and has substantial within-person variation over time. The rate of increase in BMI tended to slow down in dyslipidemic adults because they already had relatively higher BMI levels before the onset of dyslipidemia. The major strength of our individual-specific parameter analysis is to provide the potential for identification of sensitive age windows in BMI change for future health outcomes.

Marked differences in long-term levels and trajectories of BMI and lipid levels in adults were noted between race–sex groups in the present study cohort. The black–white differences in cardiovascular risk factors are well documented in previous studies [33,34,35]. Of note, the race–sex differences in BMI in black females even occurred in childhood. These race–sex differences in BMI in early life were also seen in a study of 29,254 children [35]. Despite substantial black–white and sex differences in levels of BMI and lipids, the strength of BMI–dyslipidemia associations did not differ significantly between race–sex subgroups in most models, as shown in Supplemental Table S2. We, therefore, combined the race–sex subgroups and reported the results of the total cohort.

Previous studies have reported that childhood BMI is significantly associated with adult dyslipidemia; however, the association disappeared when taking adult BMI into account [11,30]. In line with previous findings [30], we noted that adult BMI had the strongest association with the concurrent dyslipidemia, and the effect of childhood BMI was dependent on adult BMI. It is well-known that childhood BMI is closely correlated with adult BMI, and early-life obesity tends to track or persist to later life [13,14]. Existing evidence from this and previous studies suggests that the influence of excessive body weight in early life on the development of lipid disorders is mainly mediated through the strong tracking correlation of body weight during growth periods [30]. The additional information provided in this study is that the long-term impact of excessive body weight on adult dyslipidemia is amplified with increasing age from childhood.

The current longitudinal study cohorts from the i3C Consortium with large sample size and repeated BMI measurements from childhood provided a unique opportunity to characterize the life-course individual-level trajectories of BMI. This study also has certain limitations. First, although BMI is widely used as a measure to diagnose obesity, it cannot discriminate fat mass and lean mass. Second, continuous lipid variables in adults were not analyzed due to lipid-lowering medications. Third, adult BMI was included in the growth curve model fitting to calculate the life-time cumulative burden of BMI. The stronger cross-sectional association at the last adult point raised the associations of the long-term burden and trajectories of BMI with lipids.

In summary, the longitudinal cohorts of the i3C Consortium demonstrated that adults with dyslipidemia had consistently higher levels and distinct life-course trajectories of BMI from childhood to adulthood compared with those with normal lipids. Although it is still not possible to precisely diagnose which children are at risk for adult dyslipidemia, elevated BMI and its increasing trends are a life-long burden for adult dyslipidemia. Adults with dyslipidemia had higher long-term BMI levels and greater linear slopes and change in slopes compared to those with normal lipids levels. The consistent and strong associations of elevated BMI in early life and its longitudinal burden with adult dyslipidemia observed in this study support the notion that dyslipidemia is the most common comorbidity associated with obesity. The findings underscore the importance of life-long weight control, beginning in childhood, in improving lipid profiles and thus reducing future cardiovascular risk.

## Figures and Tables

**Table 1:** Characteristics of study participants.

Variable	BHS (*n* = 1739)	YFS (*n* = 1718)	MUSC (*n* = 776)	NGHS (*n* = 575)	PHBPC (*n* = 387)	Total (*n* = 5195)
Male, *n* (%)	765 (44.0)	765 (44.5)	350 (45.1)	0 (0.0)	189 (48.8)	2069 (39.8)
White, *n* (%)	1181 (67.9)	1718 (100.0)	776 (100.0)	258 (44.9)	279 (72.1)	4212 (80.1)
**Childhood (Fist exam)**						
Age (year)	10.2 (3.1)	9.7 (4.1)	12.0 (2.2)	10.0 (0.5)	7.7 (0.7)	10.1 (3.2)
BMI (kg/m^2^)	17.8 (3.5)	17.2 (2.6)	19.0 (3.2)	18.3 (3.6)	16.5 (2.2)	17.7 (3.2)
**Adulthood (Last exam)**						
Age (year)	38.5 (7.1)	39.5 (4.4)	39.9 (8.7)	26.2 (0.9)	39.1 (1.9)	37.7 (7.2)
BMI (kg/m^2^)	29.4 (7.6)	26.2 (4.7)	27.8 (6.1)	28.4 (7.9)	29.2 (6.9)	28.0 (6.7)
Overweight, *n* (%) a	526 (30.3)	621 (36.2)	235 (30.3)	134 (23.3)	118 (30.5)	1634 (31.5)
Obesity, *n* (%) b	673 (38.7)	326 (19.0)	251 (32.4)	200 (34.8)	150 (38.8)	1600 (30.8)
LDL-C (mg/dL)	126.1 (33.4)	124.8 (31.6)	117.5 (33.1)	101.4 (30.7)	111.5 (29.2)	120.6 (33.2)
HDL-C (mg/dL)	48.3 (14.6)	50.8 (12.3)	49.8 (14.4)	51.5 (13.2)	50.4 (13.8)	49.9 (13.7)
TG (mg/dL)	131.1 (96.0)	110.7 (57.3)	110.5 (66.0)	95.6 (51.0)	116.2 (65.8)	116.2 (74.6)
High LDL-C, *n* (%)	343 (19.7)	251 (14.6)	122 (15.7)	26 (4.5)	34 (8.8)	776 (14.9)
Low HDL-C, *n* (%)	568 (32.7)	357 (20.8)	232 (29.9)	102 (17.7)	96 (24.8)	1355 (26.1)
High TG, *n* (%)	337 (19.4)	179 (10.4)	123 (15.9)	26 (4.5)	54 (14.0)	719 (13.8)
Dyslipidemia, *n* (%)	794 (45.7)	569 (33.1)	300 (38.7)	132 (22.8)	121 (31.3)	1916 (36.9)
**AUC measures**						
Average age (year)	22.5 (4.4)	23.4 (4.4)	26.4 (6.3)	17.6 (0.8)	14.9 (0.7)	22.3 (5.3)
BMI AUCt (kg/m^2^)	24.7 (5.1)	22.7 (3.3)	24.5 (4.2)	24.5 (5.5)	24.7 (4.7)	24.0 (4.6)
BMI AUCi (kg/m^2^)	6.9 (3.9)	5.6 (2.3)	5.3 (3.1)	5.9 (3.2)	8.5 (3.7)	6.2 (3.3)

Values are mean (SD) and *n* (%). BHS = Bogalusa Heart Study; YFS = Cardiovascular Risk in Young Finns Study; MUSC = Muscatine Study; NGHS = NHLBI Growth and Health Study; PHBPC = Prevention of High Blood Pressure in Children; BMI = body mass index; LDL-C = low-density lipoprotein cholesterol; HDL-C = high-density lipoprotein cholesterol; TG = triglycerides; AUC_t_ = total area under the curve; AUC_i_ = incremental area under the curve. ^a^ Overweight was defined as BMI ≥25 kg/m^2^ and <30 kg/m^2^; ^b^ Obesity was defined as BMI ≥ 30 kg/m^2^.

**Table 2:** Growth curve parameters of BMI in means (SDs) between dyslipidemia groups.

Curve Parameters	Dyslipidemia	High LDL-C	Low HDL-C	High TG
*n* (yes/no)	1916/3279	776/4419	1355/3840	719/4476
β_0_ + b_0_ (kg/m^2^)				
Yes	25.3 (5.3)	24.9 (4.8)	26.0 (5.5)	25.4 (5.0)
No	23.5 (4.4)	24.1 (4.8)	23.6 (4.4)	24.0 (4.8)
P a	<0.001	<0.001	<0.001	<0.001
β_1_ + b_1_ (kg/m^2^/year)				
Yes	0.45 (0.26)	0.43 (0.24)	0.47 (0.27)	0.48 (0.24)
No	0.32 (0.26)	0.35 (0.27)	0.33 (0.26)	0.35 (0.27)
*p* ^a^	<0.001	<0.001	<0.001	<0.001
β_2_ + b_2_ (10 kg/m^2^/year^2^) ^b^				
Yes	−0.101 (0.100)	−0.091 (0.087)	−0.108 (0.104)	−0.093 (0.095)
No	−0.106 (0.096)	−0.107 (0.099)	−0.103 (0.094)	−0.106 (0.098)
*p* ^a^	0.043	<0.001	0.155	<0.001
β_3_ + b_3_ (20 kg/m^2^/year^3^) ^b^				
Yes	0.002 (0.010)	0.002 (0.009)	0.002 (0.010)	0.001 (0.009)
No	0.005 (0.011)	0.004 (0.011)	0.004 (0.010)	0.004 (0.011)
*p* ^a^	<0.001	<0.001	<0.001	<0.001

BMI = body mass index; LDL-C = low-density lipoprotein cholesterol; HDL-C = high-density; lipoprotein cholesterol; TG = triglycerides; Dyslipidemia was defined as any of high LDL-C, low HDL-C or high TG; ^a^ *p*-values for difference in βs + bs between dyslipidemia and normal groups were adjusted for average age, race, sex and cohort, with additional adjustment for β_0_ + b_0_ for other curve parameters; ^b^ β_2_ + b_2_ and β_3_ + b_3_ were estimated using the terms of age^2^/10 and age^3^/20, respectively, for model fitting.

**Table 3:** Standardized odds ratios (ORs) and 95% confidence intervals (CIs) of BMI measures for adult dyslipidemia.

Independent Variable	Dependent Variable			
	Dyslipidemia	High LDL-C	Low HDL-C	High TG
Childhood BMI ^a^	1.22 (1.15–1.29)	1.10 (1.02–1.19)	1.29 (1.22–1.37)	1.13 (1.05–1.22)
Adulthood BMI	1.85 (1.74–1.97)	1.42 (1.32–1.53)	1.82 (1.71–1.95)	1.65 (1.53–1.77)
BMI AUC_t_^b^	1.61 (1.52–1.71)	1.30 (1.21–1.40)	1.68 (1.57–1.79)	1.48 (1.38–1.59)
BMI AUC_i_^c^	1.59 (1.50–1.69)	1.30 (1.21–1.40)	1.62 (1.52–1.73)	1.50 (1.40–1.62)

ORs are all significantly greater than 1 (*p* < 0.01). BMI = body mass index; LDL-C = low-density lipoprotein cholesterol; HDL-C = high-density lipoprotein cholesterol; TG = triglycerides; AUC_t_ = total area under the curve; AUC_i_ = incremental area under the curve; Dyslipidemia was defined as any of high LDL-C, low HDL-C or high TG. Covariates included adult age, race, sex and cohort. ORs of the four BMI measures were estimated in separate models; ^a^ adjusted for childhood age prior to regression analyses; ^b^ adjusted for average age prior to regression analyses; ^c^ adjusted for average age and childhood BMI prior to regression analyses.

## Supplementary Materials

The following are available online at https://www.mdpi.com/2077-0383/8/10/1725/s1, Figure S1: The area under the curve (AUC) of body mass index (BMI), Figure S2: Growth curves of body mass index (BMI) by adult dyslipidemia in race-sex groups, Figure S3: Odds ratio (OR) and 95% confidence interval (CI) for adult dyslipidemia by childhood and adulthood BMI groups, adjusting for race, sex, age and cohort, Table S1: Characteristics of included and excluded participants, Table S2: Characteristics of participants by race and sex, Table S3: Standardized odds ratios (ORs) and 95% confidence intervals (CIs) of BMI measures for adult dyslipidemia by race and sex, Table S4: Standardized odds ratios (ORs) and 95% confidence intervals (CIs) of BMI measures for adult dyslipidemia by adult age groups, Table S5: Standardized odds ratios (ORs) and 95% confidence intervals (CIs) of BMI burden for adult dyslipidemia with and without adjusting for the last adult BMI.
