# Supplementary material for: Long-Term Burden of Increased Body Mass Index from Childhood on Adult Dyslipidemia: The i3C Consortium Study
Source: J Clin Med. 2019 Oct 18;8(10):1725. doi: 10.3390/jcm8101725 (PMC6832308; doi:10.3390/jcm8101725)
Supplement: Supplementary file 1 [file jcm-08-01725-s001.pdf]

Supplemental Materials

Study Cohorts

The Bogalusa Heart Study

The Bogalusa Heart Study (BHS), a series of long-term observations in a semi-rural biracial (65% white and 35% black) community in Bogalusa, Louisiana, was founded by Dr. Gerald Berenson in 1973. It is focused on understanding the early natural history of cardiovascular disease and childhood risk factors. In the community of Bogalusa, Louisiana, 9 surveys of children aged 4-18 years and 11 surveys of adults aged 19-51 years who were previously examined as children were conducted between 1973 and 2010. The last survey was conducted in 2007-10, with recruitment of 914 adult participants aged 36-52 years.

The Cardiovascular Risk in Young Finns Study

The Cardiovascular Risk in Young Finns Study (YFS) is a population-based multicenter study in Finland and the largest European CV risk factor follow-up study from childhood to adulthood. Participants aged 3-18 years (N=3596) were recruited in 1980, and this cohort has been followed-up every 3-6 years. The most recent in person examination was in 2011 with participation of 2041 individuals (62% of the 3318 invited).

The Muscatine Study

The Muscatine Study (MUSC) was initiated in 1970 in the schools of Muscatine, Iowa. Approximately 70% of the eligible school population (11,377 students, 5-18 years at baseline) had data collected over the following 12 years. During 1982-91, a representative subset of 2547 (age 20-39) was re-examined, and a subsample of those (n=906) was followed longitudinally between 1992-2008.

The NHLBI Growth and Health Study

The NHLBI Growth and Health Study (NGHS) was started in 1987 as a longitudinal fixed-cohort study of girls (9-10 years at baseline and followed annually for 10 years to age 19) at three clinical centers (Richmond, CA, Cincinnati, OH, and Washington, DC). Only the Cincinnati center (N=870) is participating in the i3C Consortium. Yearly examinations were conducted between ages 19-24, and there were two visits between ages 25-29. The last in-person examination was conducted between 2003-06, with recruitment of 535 participants.

The Prevention of High BP in Children Study

The Prevention of High BP in Children study (PHBPC) was initiated in 1977-78 with BP screening of 10,423 6-8 year olds. A cohort of 1207 was selected for long-term evaluation, with stratification for race. They were seen twice yearly through grade school, once yearly through high school, and at ages 19 and 23. At age 39, 81% of a limited group of 480 individuals with children were found for a comprehensive anthropometric and metabolic screening.

Table S1. Characteristics of included and excluded participants.

	Included (N=5195)	Excluded (N=4535)	P
Male, n (%)	2069 (39.8)	2170 (47.9)	<0.001
White, n (%)	4212 (80.1)	3703 (81.7)	<0.001
**Childhood (First exam)**			
Age (yr)	10.1 (3.3)	11.6 (4.4)	<0.001
BMI (kg/m^2)	17.7 (3.2)	18.8 (3.9)	<0.001
**Adulthood (Last exam)**			
Age (yr)	34.1 (6.8)	29.8 (8.4)	<0.001
BMI (kg/m^2)	28.0 (6.7)	26 (6.1)	<0.001
LDL-C (mg/dL) [a]	120.6 (33.2)	114.7 (33.1)	<0.001
HDL-C (mg/dL) [a]	49.9 (13.7)	49.8 (13.7)	0.690
TG (mg/dL) [a, b]	116.2 (74.6)	106.9 (75.7)	<0.001
High LDL-C, n (%)	776 (14.9)	420 (9.3)	<0.001
Low HDL-C, n (%)	1355 (26.1)	1074 (23.7)	0.006
High TG, n (%)	719 (13.8)	401 (8.8)	<0.001
Dyslipidemia, n (%)	1916 (36.7)	1445 (31.9)	<0.001

Values are mean (SD) and n (%). BMI=body mass index; LDL-C=low-density lipoprotein cholesterol; HDL-C=high-density lipoprotein cholesterol; TG=triglycerides; a, Participants who were on medication were excluded; b, Non-fasting samples were excluded.

Table S2. Characteristics of participants by race and sex.

Study variable	White		Black		P for race difference	
	Male (n=1796)	Female (n=2416)	Male (n=273)	Female (n=710)	Male	Female
**Childhood (First exam)**						
Age (yr)	10.2 (3.4)	10.1 (3.2)	10.0 (2.9)	9.8 (2.1)	0.540	0.001
BMI (kg/m^2)	17.8 (3.1)	17.6 (3.1)	17.3 (3.2)	18.0 (3.7)*	0.054	0.011
**Adulthood (Last exam)**						
Age (yr)	39.5 (6.1)	37.9 (7.3)*	38.4 (7.0)	32.7 (7.8)*	0.021	<0.001
BMI (kg/m^2)	28.1 (5.3)	26.8 (6.5)*	29.3 (7.5)	31.1 (8.5)*	0.010	<0.001
LDL-C (mg/dL) [a]	129.6 (33.3)	117.0 (31.3)*	119.6 (35.2)	110.4 (32.5)*	<0.001	<0.001
HDL-C (mg/dL) [a]	44.5 (11.1)	53.5 (13.6)*	50.0 (17.7)	51.4 (14.3)	<0.001	0.002
TG (mg/dL) [a, b]	139.5 (90.7)	105.8 (59.8)*	126.2 (89.1)	89.0 (45.8)*	0.023	<0.001
High LDL-C, n (%)	397 (22.1)	268 (11.1)*	45 (16.5)	70 (9.5)*	0.035	0.173
Low HDL-C, n (%)	739 (41.2)	394 (16.3)*	84 (30.8)	138 (19.4)*	<0.001	0.051
High TG, n (%)	395 (22.0)	242 (10.0)*	44 (16.1)	38 (5.4)*	0.027	<0.001
Dyslipidemia, n (%)	976 (54.3)	637 (26.4)*	121 (44.3)	182 (25.6)*	0.002	0.697
**AUC measures**						
Average age (yr)	23.2 (5.5)	22.6 (5.3)*	21.1 (4.8)	19.4 (3.9)*	<0.001	<0.001
BMI AUC_t (kg/m^2)	24.1 (3.9)	23.2 (4.3)*	24.6 (5.0)	26.0 (6.0)*	0.161	<0.001
BMI AUC_i (kg/m^2)	6.4 (2.7)	5.6 (3.3)*	7.2 (3.5)	7.9 (4.3)*	<0.001	<0.001

Values are mean (SD) and n (%). BMI=body mass index; LDL-C=low-density lipoprotein cholesterol; HDL-C=high-density lipoprotein cholesterol; TG=triglycerides; AUC_t=total area under the curve; AUC_i=incremental area under the curve; a, Participants who were on medication were excluded; b, Nonfasting samples were excluded. * P<0.01 for sex difference within racial groups.

Table S3. Standardized odds ratios (ORs) and 95% confidence intervals (CIs) of BMI measures for adult dyslipidemia by race and sex.

Independent Variable	White		Black		P for race difference	
	Male	Female	Male	Female	Male	Female

**Dependent Variable: Dyslipidemia**						
Childhood BMI [a]	1.09 (0.99-1.21)	1.36 (1.24-1.49)*	1.26 (1.01-1.58)	1.25 (1.09-1.42)	0.157	0.439
Adulthood BMI	2.27 (1.97-2.62)	2.01 (1.83-2.22)	1.76 (1.39-2.23)	1.61 (1.41-1.83)	0.098	0.003
BMI AUC_t [b]	1.66 (1.46-1.88)	1.84 (1.67-2.02)	1.61 (1.27-2.05)	1.46 (1.29-1.65)	0.831	0.003
BMI AUC_i [c]	1.95 (1.71-2.24)	1.66 (1.51-1.82)	1.61 (1.26-2.06)	1.41 (1.24-1.61)	0.607	0.030
**Dependent Variable: High LDL-C**						
Childhood BMI [a]	1.01 (0.90-1.14)	1.23 (1.08-1.39)*	1.13 (0.86-1.47)	1.12 (0.91-1.36)	0.455	0.867
Adulthood BMI	1.55 (1.35-1.78)	1.46 (1.31-1.64)	1.46 (1.13-1.89)	1.28 (1.07-1.53)	0.800	0.197
BMI AUC_t [b]	1.30 (1.14-1.48)	1.38 (1.23-1.55)	1.42 (1.10-1.83)	1.20 (1.01-1.43)	0.546	0.211
BMI AUC_i [c]	1.46 (1.28-1.67)	1.29 (1.15-1.44)	1.46 (1.11-1.92)	1.14 (0.95-1.37)	0.988	0.157
**Dependent Variable: Low HDL-C**						
Childhood BMI [a]	1.15 (1.04-1.28)	1.50 (1.36-1.67)*	1.33 (1.06-1.67)	1.33 (1.15-1.53)	0.177	0.162
Adulthood BMI	2.01 (1.76-2.30)	2.02 (1.82-2.24)	1.86 (1.46-2.37)	1.76 (1.53-2.03)	0.556	0.092
BMI AUC_t [b]	1.64 (1.46-1.86)	1.98 (1.78-2.20)*	1.74 (1.36-2.22)	1.60 (1.40-1.83)	0.679	0.015
BMI AUC_i [c]	1.80 (1.59-2.05)	1.74 (1.57-1.93)	1.71 (1.32-2.20)	1.55 (1.35-1.78)	0.620	0.242
**Dependent Variable: High TG**						
Childhood BMI [a]	1.15 (1.03-1.29)	1.12 (0.98-1.28)	1.25 (0.97-1.62)	1.10 (0.85-1.43)	0.488	0.901
Adulthood BMI	2.17 (1.87-2.5)	1.70 (1.51-1.91)*	1.39 (1.07-1.82)	1.25 (0.99-1.58)	0.005	0.011
BMI AUC_t [b]	1.71 (1.50-1.95)	1.53 (1.36-1.72)	1.42 (1.10-1.85)	1.29 (1.04-1.61)	0.247	0.269
BMI AUC_i [c]	1.83 (1.59-2.11)	1.55 (1.38-1.73)	1.30 (0.98-1.72)	1.31 (1.06-1.64)	0.089	0.251

BMI=body mass index; LDL-C=low-density lipoprotein cholesterol; HDL-C=high-density lipoprotein cholesterol; TG=triglycerides; AUC_t=total area under the curve; AUC_i=incremental area under the curve. ORs of the four BMI measures were estimated in separate models. Covariates included adult age and cohort; a, adjusted for childhood age prior to regression analyses; b, adjusted for avererage age prior to regression analyses; c, adjusted for avererage age and childhood BMI prior to regression analyses; * P<0.05 for sex difference within racial groups.

Table S4. Standardized odds ratios (ORs) and 95% confidence intervals (CIs) of BMI measures for adult dyslipidemia by adult age groups.

**Adult age groups**	Prevalence of dyslipidemia (%)	Independent variable			
		Childhood BMI [a]	Adulthood BMI	BMI AUC_t [b]	BMI AUC_i [c]
≤30 yr (n=1159)	27.8	1.34 (1.08-1.66)	1.70 (1.38-2.11)	1.59 (1.29-1.96)	1.47 (1.15-1.87)
31-35 yr (n=716)	33.1	1.41 (1.07-1.86)	2.06 (1.6-2.64)	1.92 (1.49-2.49)	1.76 (1.39-2.23)
36-40 yr (n=1480)	37.6	1.14 (0.97-1.33)	1.79 (1.54-2.09)	1.55 (1.34-1.80)	1.51 (1.31-1.74)
41-45 yr (n=1066)	41.9	1.11 (0.96-1.30)	1.67 (1.43-1.96)	1.46 (1.25-1.70)	1.59 (1.35-1.87)
≥46 yr (n=774)	46.6	1.03 (0.89-1.18)	1.61 (1.36-1.91)	1.39 (1.18-1.63)	1.55 (1.30-1.85)

BMI=body mass index; LDL-C=low-density lipoprotein cholesterol; HDL-C=high-density lipoprotein cholesterol; TG=triglycerides; AUC_t=total area under the curve; AUC_i=incremental area under the curve; Dyslipidemia was defined as any of high LDL-C, low HDL-C or high TG. Covariates included

adult age, race, sex and cohort. ORs of the four BMI measures were estimated in separate models; a, adjusted for childhood age prior to regression analyses; b, adjusted for average age prior to regression analyses; c, adjusted for average age and childhood BMI prior to regression analyses.

Table S5. Standardized odds ratios (ORs) and 95% confidence intervals (CIs) of BMI burden for adult dyslipidemia with and without adjusting for the last adult BMI.

Independent variable	Dependent Variable			
	**Dyslipidemia**	**High LDL-C**	**Low HDL-C**	**High TG**
Model 1:				
BMI burden	1.57 (1.47-1.67) **	1.27 (1.18-1.37) **	1.67 (1.56-1.78) **	1.43 (1.33-1.55) **
Model 2:				
Last BMI	1.85 (1.74-1.97) **	1.42 (1.32-1.53) **	1.82 (1.71-1.95) **	1.65 (1.53-1.77) **
Model 3:				
BMI burden	0.73 (0.65-0.82) **	0.78 (0.68-0.90) **	0.88 (0.79-0.99) *	0.77 (0.66-0.89) **
Last adult BMI	2.60 (2.31-2.93) **	1.79 (1.56-2.05) **	2.20 (1.95-2.49) **	2.16 (1.86-2.50) **

* $P<0.05$, ** $P<0.01$ for OR being significantly different from 1. BMI=body mass index; LDL-C=low-density lipoprotein cholesterol; HDL-C=high-density lipoprotein cholesterol; TG=triglycerides. Dyslipidemia was defined as any of high LDL-C, low HDL-C or high TG. Covariates included adult age, race, sex and cohort. BMI burden was calculated as the mean of regression residuals of BMI at all the age-points before the last adult BMI measurement.

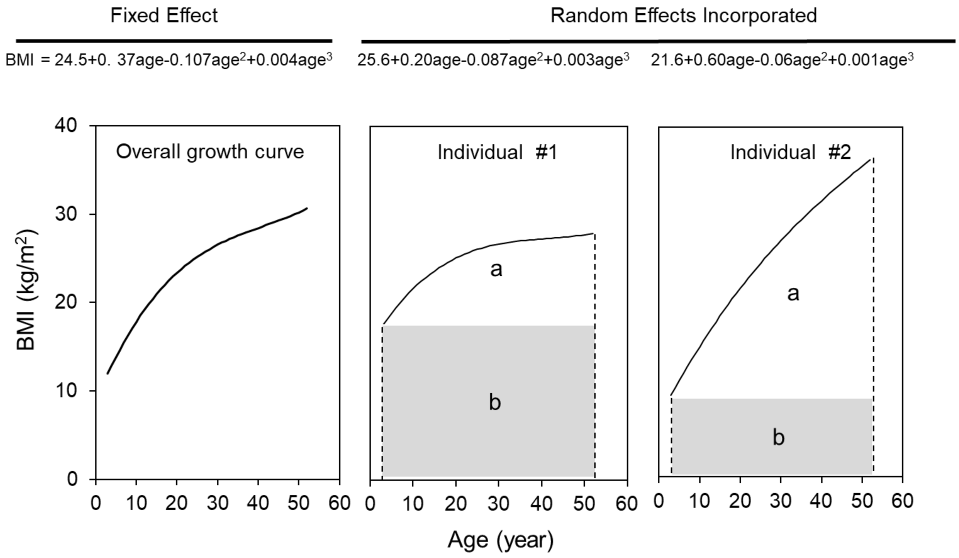

Supplemental Figure 1. The area under the curve (AUC) of body mass index (BMI)
a = incremental AUC; b = baseline AUC; a+b = total AUC

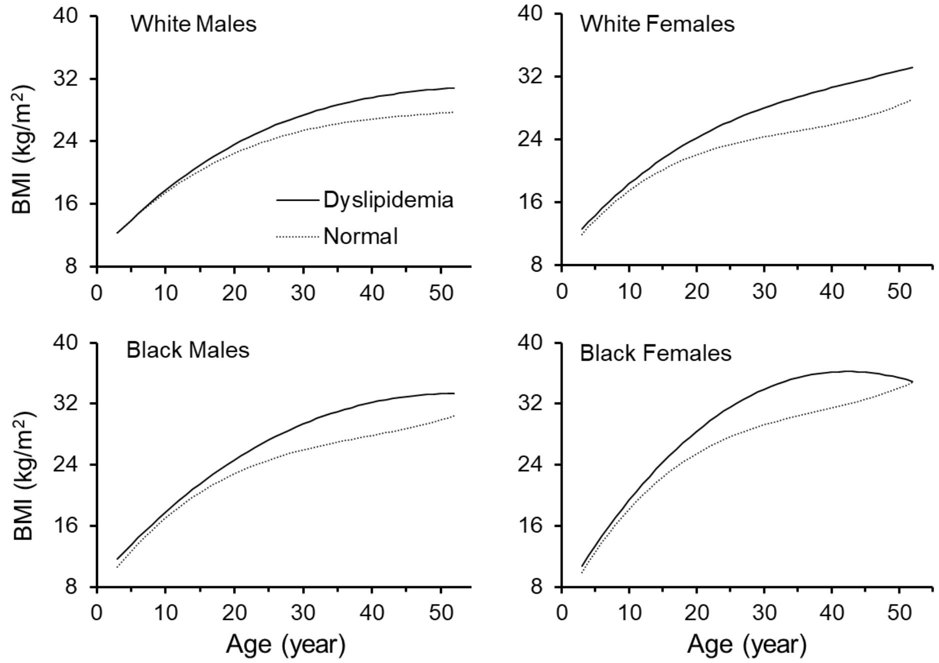

Supplemental Figure 2. Growth curves of body mass index (BMI) by adult dyslipidemia in race-sex groups

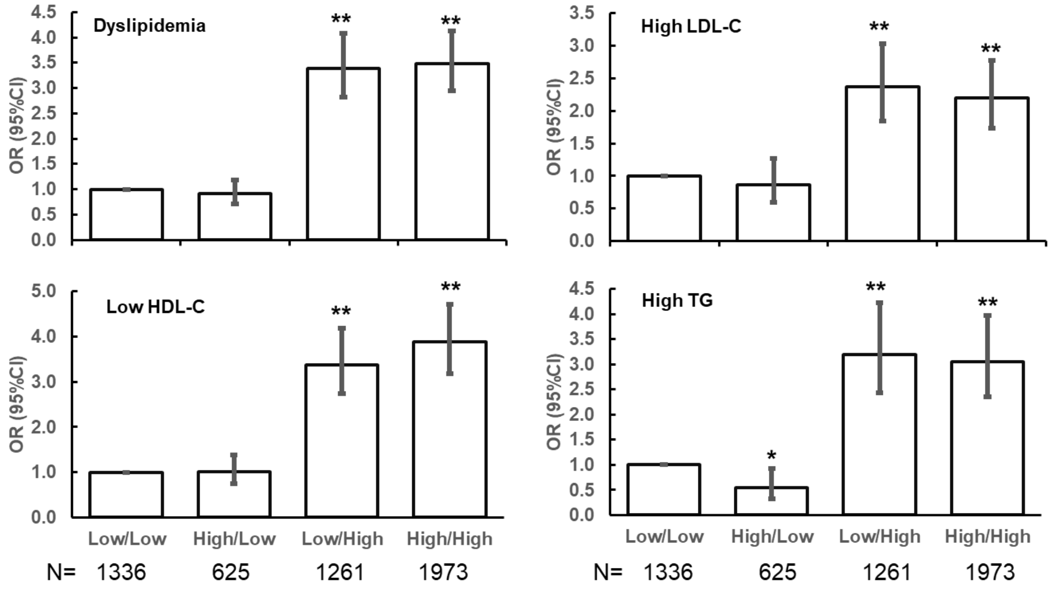

Supplemental Figure 3. The Odds ratios of lipid disorders according to childhood and adulthood BMI status adjusting for race, sex, age and cohort

Low/Low, childhood low BMI and adult low BMI; High/Low, childhood high BMI and adult low BMI; Low/High, childhood low BMI and adult high BMI; High/High, childhood high BMI and adult high BMI.

High/low BMI in childhood was defined by its race-sex specific medians; high/low BMI in adulthood was defined by 25 kg/m^2.

Compared with low/low group: * P<0.01, **P<0.001.
